# The public health and mental health implications of alcohol consumption: a focus on epidemiology and prevention strategies

**DOI:** 10.1192/j.eurpsy.2025.740

**Published:** 2025-08-26

**Authors:** M. Bivol, J. Chihai

**Affiliations:** 1 Department of Mental Health, Medical Psychology and Psychotherapy, Nicolae Testemitanu State University of Medicine and Pharmacy of the Republic of Moldova, Chisinau, Moldova, Republic of

## Abstract

**Introduction:**

Alcohol use remains a significant public health issue across Europe, contributing to approximately 5% of premature deaths and 25% of all fatal road accidents. The effects of excessive alcohol consumption are widespread, impacting both physical and mental health. In Moldova, as in many other regions, alcohol use is a contributing factor to a range of health issues, including alcohol dependence and alcohol-induced psychoses. This underscores the importance of comprehensive strategies to address these challenges and promote better mental health outcomes.

**Objectives:**

This study aims to analyze the epidemiological trends of alcohol use in Europe, particularly Moldova, and its associated mental health consequences. It explores the role of alcohol in the development of psychiatric comorbidities, including depression and anxiety, and discusses strategies for prevention and treatment.

**Methods:**

Data from WHO and local health authorities were used to assess the incidence and prevalence of alcohol dependence and alcohol-induced psychoses. The study tracked trends from 2019 to 2023 across different regions in Moldova, comparing them with European data. Furthermore, the study examined the links between alcohol use and mental health disorders like depression and bipolar disorder, highlighting the complexities of managing these co-occurring conditions.

**Results:**

The analysis of alcohol use patterns in Moldova revealed significant regional variations in both alcohol dependence and alcohol-induced psychoses. For instance, the Northern region experienced an increase in alcohol dependence rates from 79,2 cases per 100,000 in 2019 to 99,2 in 2023. Similarly, the prevalence of alcohol-induced psychoses in the Central region rose from 26,4 cases per 100,000 in 2019 to 35,2 in 2023. These patterns are consistent with trends observed across Europe, where alcohol consumption is frequently linked to heightened risks of developing mental health disorders, particularly depression and anxiety. Furthermore, alcohol-related psychiatric conditions pose challenges for effective treatment, often requiring integrated care that addresses both substance use and mental health.

**Conclusions:**

The evolving patterns of alcohol use and its associated mental health consequences highlight the need for integrated, non-stigmatizing public health interventions. Prevention strategies that combine public health education, policy reform, and improved access to mental health services can help reduce the burden of alcohol use on individuals and communities. Early intervention and personalized care approaches are essential for mitigating the dual impact of alcohol and mental health issues.

**Disclosure of Interest:**

None Declared

